# Attenuation of Hind-Limb Ischemia in Mice with Endothelial-Like Cells Derived from Different Sources of Human Stem Cells

**DOI:** 10.1371/journal.pone.0057876

**Published:** 2013-03-05

**Authors:** Wing-Hon Lai, Jenny C. Y. Ho, Yau-Chi Chan, Joyce H. L. Ng, Ka-Wing Au, Lai-Yung Wong, Chung-Wah Siu, Hung-Fat Tse

**Affiliations:** 1 Cardiology Division, Department of Medicine, Queen Mary Hospital, the University of Hong Kong, Hong Kong, HKSAR, China; 2 Research Center of Heart, Brain, Hormone and Healthy Aging, Li Ka Shing Faculty of Medicine, the University of Hong Kong, Hong Kong, HKSAR, China; University of Illinois at Chicago, United States of America

## Abstract

Functional endothelial-like cells (EC) have been successfully derived from different cell sources and potentially used for treatment of cardiovascular diseases; however, their relative therapeutic efficacy remains unclear. We differentiated functional EC from human bone marrow mononuclear cells (BM-EC), human embryonic stem cells (hESC-EC) and human induced pluripotent stem cells (hiPSC-EC), and compared their *in-vitro* tube formation, migration and cytokine expression profiles, and *in-vivo* capacity to attenuate hind-limb ischemia in mice. Successful differentiation of BM-EC was only achieved in 1/6 patient with severe coronary artery disease. Nevertheless, BM-EC, hESC-EC and hiPSC-EC exhibited typical cobblestone morphology, had the ability of uptaking DiI-labeled acetylated low-density-lipoprotein, and binding of *Ulex europaeus* lectin. *In-vitro* functional assay demonstrated that hiPSC-EC and hESC-EC had similar capacity for tube formation and migration as human umbilical cord endothelial cells (HUVEC) and BM-EC (*P>0.05*). While increased expression of major angiogenic factors including epidermal growth factor, hepatocyte growth factor, vascular endothelial growth factor, placental growth factor and stromal derived factor-1 were observed in all EC cultures during hypoxia compared with normoxia (*P<0.05*), the magnitudes of cytokine up-regulation upon hypoxic were more dramatic in hiPSC-EC and hESC-EC (*P<0.05*). Compared with medium, transplanting BM-EC (n = 6), HUVEC (n = 6), hESC-EC (n = 8) or hiPSC-EC (n = 8) significantly attenuated severe hind-limb ischemia in mice via enhancement of neovascularization. In conclusion, functional EC can be generated from hECS and hiPSC with similar therapeutic efficacy for attenuation of severe hind-limb ischemia. Differentiation of functional BM-EC was more difficult to achieve in patients with cardiovascular diseases, and hESC-EC or iPSC-EC are readily available as “off-the-shelf” format for the treatment of tissue ischemia.

## Introduction

In experimental studies [1] and clinical trials [2]–[4], human bone marrow (BM) derived stem cells have been used as potential cell therapy to enhance angiogenesis in ischemic tissue, however, their therapeutic application are limited by the ability to obtain sufficient number of stem cells for transplantation. Furthermore, aging and underlying cardiovascular risk factors [5], such as diabetes and heart failure [6] in patients with cardiovascular diseases affect the number and capacity of their circulating BM-derived progenitor cells [7], [8] which further hamper the effectiveness of autologous BM cell therapy.

Prior studies [9] have demonstrated that functional endothelial-like cells (EC) can be derived from pluripotent human embryonic stem cells (hESC) for therapeutic angiogenesis. Nonetheless, the clinical application of hESC-EC is limited by the ethical issue related to the generation of hESC as well as the potential risk of immune rejection. Recent advances in the generation of human induced pluripotent stem cells (hiPSC) [10], [11], [12] may represent an alternative source to derive functional EC for treatment of cardiovascular diseases. As hiPSC can be generated in a patient-specific manner, which is genetically identical to individual patients, they can avoid the potential immune rejections after transplantation as well as the ethical concerns on generating hESC [11]. Indeed, functional EC have been successfully derived from hiPSC [13]. However, the therapeutic efficacy of hiPSC derived EC for angiogenesis as compared with hESC-EC or BM stem cells derived EC have not been studied. Here, we compared the *in-vitro* and *in-vivo* angiogenic effects of EC derived from hiPSC, hESC and human BM mononuclear cells.

## Methods

### Ethics Statement

The patient study was approved by the Institutional Review Board of the University of Hong Kong/Hospital Authority Hong Kong West Cluster (HKCTR-725, http://www.hkclinicaltrials.com), and all subjects provided written informed consent. The animal study protocol conforms to the Guide for the Care and Use of Laboratory Animals published by the United States National Institutes of Health and was approved by the ethics committee of the University of Hong Kong (1896–09).

### Maintenance and Differentiation of Functional Endothelial-like Cells

Undifferentiated hiPSC: IMR90-iPSC (hiPSC-1, passage 15–25) and KS1-iPS (hiPSC-2, passage 15–25) [14] and hESC line: H1 (passage 40–50, WiCell Research Institute, Madison, WI) were maintained on Matrigel™ (BD Biosciences, MA)-coated dishes with mTeSR-1™ medium (Stem Cell Technologies, BC, Canada). Differentiation of EC from hiPSCs and hESCs were induced using formation of embryoid bodies (EB). Briefly, cell clusters were digested by 1 mg/ml dispase (Gibco, Gaithersburg, MD) and re-suspended in differentiation medium which consists of knockout-DMEM with 20% fetal calf serum (Hyclone, Logan, UT), 2 mM L-glutamine, 0.1 mM non-essential amino acids and 0.1 mM β-mercaptoethanol (Invitrogen, Carlsbad, CA) in non-coated dishes for 9 days. The resulting EB were plated on gelatin-coated 10-cm dish for 7 days. Then the central portions of the attached EBs were manually dissected out for further expansion using endothelial growth medium-2 (EGM-2, Lonza, Walkersville, MD) for 14 days. CD45^-^ CD31^+^ cells were then isolated by MoFlo XPD cell sorter (Beckman-Coulter, Fullerton, CA) and designated as hESC-EC and hiPSC-EC. For characterization, 4.8 µg/ml of DiI labeled acetylated low-density lipoprotein (DiI-AcLDL, Molecular Probes, Eugene, OR) was added and incubated at 37°C for 5 hour. Cells were washed by phosphate buffered saline, fixed in 2% paraformaldehyde for 10 minutes and then stained by 10 µg/ml of *Ulex europaeus* Lectin-FITC (Sigma Aldrich, St. Louis, MO) for 1 hour at room temperature [9]. CD31, von Willebrand factor (vWF) and intercellular adhesion molecule-1 (ICAM-1) immunofluorescence staining was performed by the endothelial cell characterization kit (Chemicon, Temecula, CA). Fluorescence-activated cell analysis (FACS) was performed with PE-labeled antibodies against CD31 (BD Bioscience, San Jose, CA), vWF (Beckman Coulter, Indianapolis, IN) and Kinase insert domain receptor (KDR; Sigma, St Louis, MO). Human umbilical cord endothelial cells (HUVEC) were cultured under standard condition in EGM-2 growth medium (Lonza, walkerville, MD) as a stable human endothelial cell control.

### Differentiation of EC from Human Bone Marrow Cells

BM-MNCs were obtained from patients recruited into the placebo arm of our previous clinical trial on the use of direct endomyocardial transplantation of BM mononuclear cells for treatment of end-staged ischemic heart diseases [15] (Table 1). From each patient, 40 ml of BM blood was obtained from right iliac crest after local anesthesia. In brief, BM mononuclear cells were isolated by Ficoll (GE Healthcare, Amersham, UK) density gradient centrifugation, and plated to gelatin-coated plate at a density of 1×10^6^ per ml in 6 wells plate with EGM2 medium. The viability of the cells at the time of harvest was greater than 95%. Attached cells were harvested at Day 14 with CD31^+^ sorting as described above and designated as BM derived endothelial-like cells (BM-EC).

**Table 1:** Clinical characteristic of the patients’ marrow MNC used in this study.

										Flow cytometry analysis				
Patients Number	Sex	Age	CAD	CHF	HT	DM	PCI	CABG	Creatinine(umol/L)	%CD34^+^	%CD3^+^	%CD11b^+^		%CD117
1	Male	61	+	+	-	-	+	-	106	1.26	37.34	21.34		2.92
2	Male	67	+	+	+	+	+	-	147	1.87	16.9	18.68		1.93
3	Male	70	+	+	+	+	+	+	104	1.32	15.66	14.41		1.29
4	Male	64	+	+	+	+	+	−	146	0.92	34.25	21		0.77
5	Male	60	+	+	+	+	+	−	162	1.49	20.81	21.67		2.01
6	Male	61	+	+	+	−	−	−	104	1.3	49.34	49.21		1.88

CAD = coronary arteries diseases; CHF = chronic heart failure; HT = hypertension; DM = diabetes mellitus; Lipid = hyperlipidemia; PCI = percutaneous coronary intervention; CABG = coronary artery bypass graft, ‘+’ = present, ‘−’ = absent.

### Angiogenic Tube Formation and Migration Assay

Tube formation of the hiPSC-1-EC, hiPSC-2-EC, hESC-EC, BM-EC and HUVEC were assessed with the *In-Vitro* Angiogenesis Assay Kit (Chemicon, Temecula, CA) with 1×10^4^ cells as described with modifications [9]. Modified Boyden Chamber assay was preformed with 1×10^4^ cells of cells (hiPSC-1-EC, hiPSC-2-EC, hESC-EC, BM-EC and HUVEC) placed in the upper chamber of the Transwell® pore Polycarbonate Membrane Insert (Corning, Lowell, MA) in EBM2 medium with 1% fetal bovine serum. The chamber was placed in a 24-well plate containing EGM2 medium with or without 50 ng/ml vascular endothelial growth factor (VEGF) as chemo-attractant and incubated at 37°C for 24 hours. Then the lower side of the filter was washed with phosphate buffered saline and fixed with 2% paraformaldehyde. Migrated cells were visualized by hematoxylin staining.

### Cytokine Release

Conditioned medium was obtained by cultivating 1×10^5^ hiPSC-1-EC, hiPSC-2-EC, hESC-EC, BM-EC and HUVEC with basal EBM2 medium (without supplement) for 5 days under normoxic (21% O_2_, 5% CO_2_) or hypoxic (0.5% O_2_, 5% CO_2_) condition using the BioSpherix OxyCycler C42 system (BioSpherix, Redfield, NY) and subjected to cytokine analysis: epidermal growth factor (EGF), basic fibroblast growth factor (FGF-B), hepatocyte growth factor (HGF), leptin, VEGF, placental growth factor (PIGF) and stromal derived factor-1 (SDF-1); adhesion molecules including, ICAM1 and vascular cell adhesion molecule (VCAM) using Procarta multiplex bead-based immunoassay kit (Affymetrix, Fremont, CA) according to manufacturer’s instructions. Data acquisition and analysis were done using Bio-Plex Manager software version 4.1.1. Cell numbers were counted after the 5 days incubation for normalization.

### *In-vivo* Functional Effects of Cell Transplantation into Animal Model of Critical Hind-Limb Ischemia

Critical hind-limb ischemia was induced in male severe combined immunodeficient mice after anesthetized with xylazine (20 mg/kg) and ketamine (100 mg/kg) by intraperitoneal injection [16]. The femoral artery was excised from its proximal origin as a branch of the external iliac artery to the distal point where it bifurcates into the saphenous and popliteal arteries. After arterial ligation, mice were immediately assigned to the following experimental groups: hiPSC-1-EC group (n = 8), hiPSC-2-EC group (n = 8), hESC-EC group (n = 8), BM-EC group (n = 6), HUVEC group (n = 6) and the medium group (medium, n = 6). In each animal, a total of 3×10^6^ cells (30 µl) or medium (EGM-2) was injected intramuscularly into 3 sites of the gracilis muscle at the medial thigh of the ischemic hind-limbs. Serial laser Doppler imaging analysis (Moor Instruments, Devon, UK) was performed to monitor the blood flow of hind-limbs immediately, at Day 7, 14, 21 and 28 after femoral artery ligation and cell-transplantation. The digital color-coded images were analyzed to quantify the blood flow in the region from the knee to the toe, the mean values of perfusion were calculated.

At Day 28, tissue from the ischemic limb was harvested and formalin fixed. Mouse monoclonal anti-vWF (Chemicon, Temecula, CA) was used to determine the capillary density on paraffin embedded tissues using standard immunostaining procedure. Arteries were stained with mouse monoclonal anti-α-smooth muscle actin antibody (Serotech, Raleigh, NC). Retention of transplanted cells was tracked by anti-human nuclear antigen antibody (HNA; Chemicon International, Temecula, CA) staining.

To monitor the cell fate after transplantation of hiPSC-EC and hESC-EC, luciferase-expressing-hiPSC-1-EC and hESC-EC was generated, cultured *in-vitro* and expanded before transplantation. In brief, luciferase gene were transduced into hiPSC-1-EC and hESC-EC by lentivirus-mediated transfer and selected in the present of 1 ug/ml puromycin, respectively. After transplanting the luciferase-expressing-hiPSC-1-EC (n = 8) and hESC-EC (n = 8) into the ischemic limb, labeled cells were tracked with the IVIS 200 Bioluminescence Imaging System (Xenogen Biosciences, Cranbury, NJ) after intraperitoneal injection of 150 mg/kg D-luciferin (Xenogen Biosciences, Cranbury, NJ) at Day 7, 14 and 28 after cell transplantation. We failed to label BM-EC as they have limited passaging capacity. Signals were analyzed with the Living Image Software (Xenogen Biosciences, Cranbury, NJ).

### Statistical Analysis

Data are expressed as mean ± SEM. Statistical comparisons were performed using Student’s *t*-test or one-way ANOVA, as appropriate. The distribution pattern of cytokines levels were highly skewed, so these variables were log-transformed to normalize their distribution before analysis. Two-way ANOVA test was performed to compare different groups with time. Post-hoc Bonferroni’s correction for multiple comparisons was used. All p values <0.05 were considered statistically significant.

## Results

### EC Differentiation

**Figure 1:**
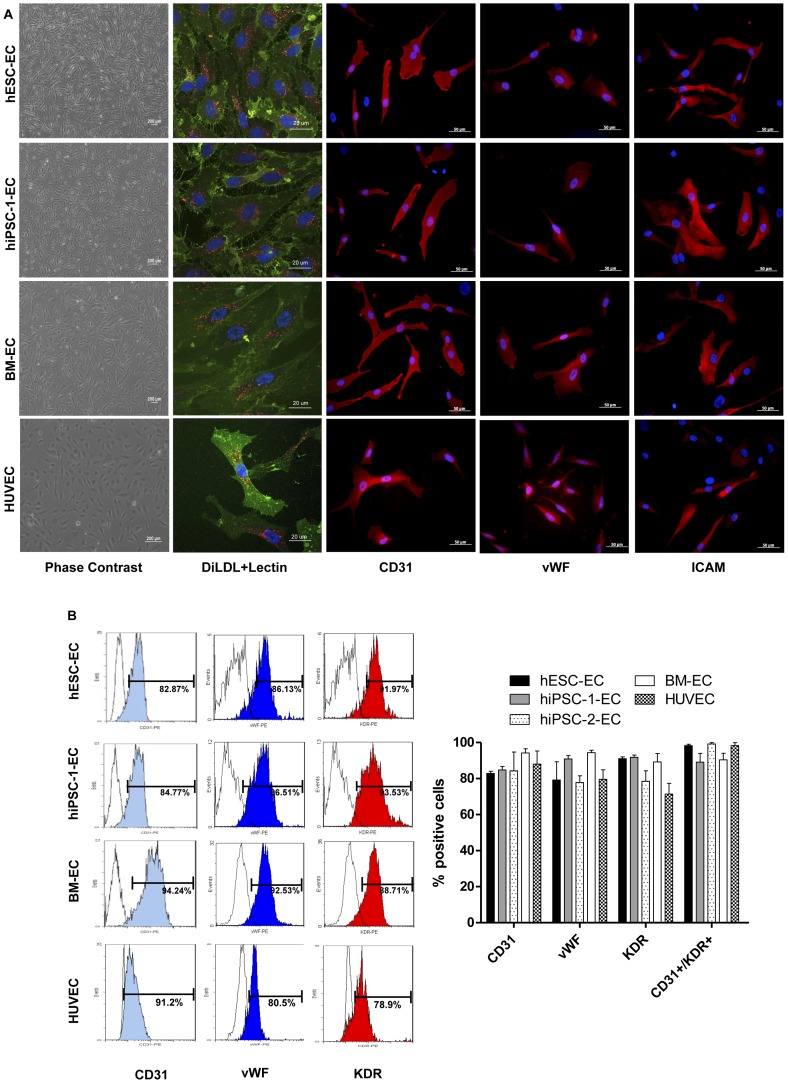
Characterization of derived endothelial-like cells (EC). (A) Phase contrast photos (left panel) showing the morphology of hESC-EC, hiPSC-EC, bone marrow derived EC (BM-EC) and human umbilical cord endothelial cell (HUVEC) in a confluent culture. Immunofluorescence staining showed the expression of typical endothelial cells markers including *Ulex europaeus* lectin (green) and uptake of Di-Acetyl-LDL (red) (second panel) in hESC-EC, hiPSC-1-EC, BM-EC and HUVEC. Furthermore, expression of CD31, von Willebrand factor (vWF) and intercellular adhesion molecule 1 (ICAM) were also detected in hESC-EC, hiPSC-EC, BM-EC and HUVEC (B) Flow cytometry analysis revealed the expression of CD31, vWF, Kinase insert domain receptor (KDR) in hESC-EC, hiPSC-EC, BM-EC and HUVEC (left panel). There were no differences in the percentage of cells with expression of CD31, vWF, KDR and co-expression of CD31 and KDR between hESC-EC, hiPSC-EC and BM-EC (right panel).

Differentiation of EC from hiPSC-1, hiPSC-2 and hESC were performed. After culturing for 3 weeks on EGM-2, EC outgrowth was observed from all hiPSC-1, hiPSC-2 and hESC (Figure 1A). BM mononuclear cells were obtained from a group of 6 patients and cultured in the EGM-2 for 14 days. However, functional EC could only be derived from 1/6 (17%) patient’s BM mononuclear cells. Culture of BM mononuclear cells obtained in the other 5 patients failed to yield any CD31^+^ cells. During the differentiation, those BM mononuclear cells from these 5 patients failed to proliferate and the majority of the cells showed nuclear fragmentation, suggesting cellular apoptosis. This finding highlights the difficulty in obtaining functional EC from patient’s autologous BM cells for transplantation.

Immunofluorescence staining showed that hiPSC-1-EC, hESC-EC and BM-EC display the characteristic phenotypes of endothelial cells, including uptake of DiI-Ac-LDL and expression of lectin as the control HUVEC (Figure 1A). Although double sorting for CD45 negative and CD31 positive was not performed in BM-EC due to the limited number of cells yielded, flow cytometry analysis revealed that only 5% of them were CD45 positive. Indeed, >90% of these differentiated EC were double positive for CD31 and KDR and >80% of them were CD31, vWF and KDR positive as determined by flow cytomety. There were no statistical significant difference in the expression of EC-surface markers between hiPSC-1-EC, hiPSC-2-EC, hESC-EC and BM-EC, and resemble to the expression in HUVEC (Figure 1B, all *P>0.05*).

### Functional Assays of EC

**Figure 2:**
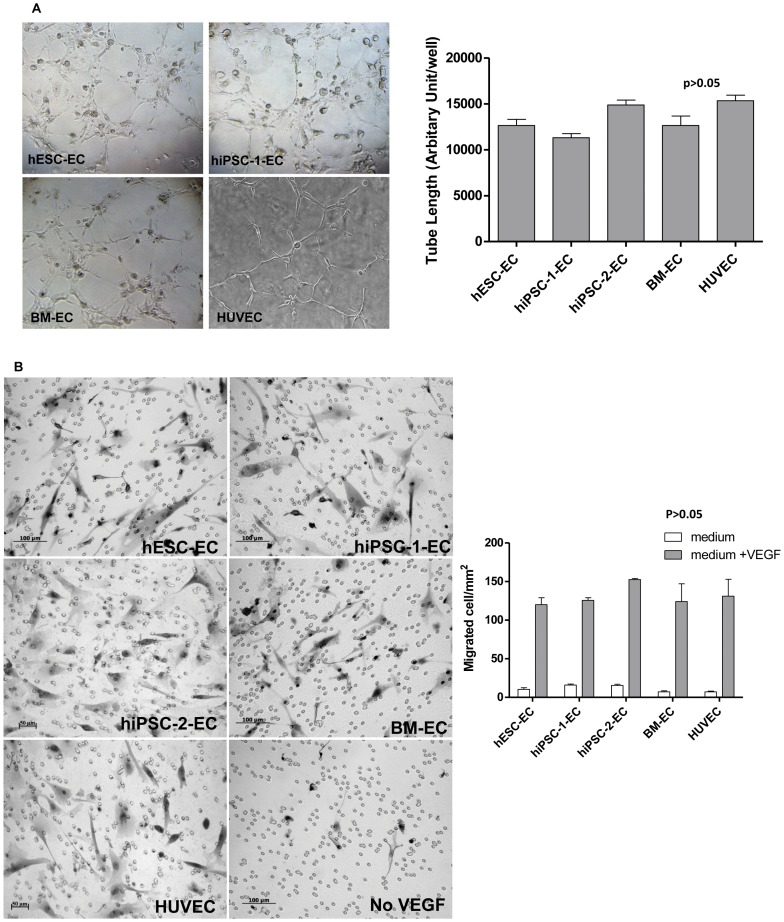
*In- vitro* functional assessment of derived endothelial-like cells. (A) Tube formation assay was performed with 1×10^4^ cells seeded onto Matrigel and the presence of micro vessel formation was visualized by phase contrast microscope (100×) after 6 hours of incubation at 37°C. There was no significant difference in length of tube-like vessels formed by hESC-EC, hiPSC-EC, BM-EC and HUVEC (p>0.05). (**B**) Modified Boyden chamber assay was used to determine the migration ability of the hESC-EC, hiPSC-EC, BM-EC and HUVEC. These cells were placed in the inner chamber and allowed to migrate across the porous membrane toward the outer chamber with the presence of medium alone (control) or medium supplemented with vascular endothelial growth factor (VEGF). Then, the numbers of migrated cells per mm^2^ present in the transwell were counted under light microscope (100×, upper panel). The numbers of migrated cells were significantly increased after supplement with VEGF in hESC-EC, hiPSC-EC, BM-EC and HUVEC as compared with their corresponding control (*p<0.05). However, there were no differences among those EC in the number of migrated cells with the presence of medium alone or medium supplemented with VEGF (p<0.05, lower panel).

The *in-vitro* functional capacity of EC was examined by their ability to form capillary like network on Matrigel in the tube-formation assay. CD31^+^ hiPSC-EC, hESC-EC, BM-EC, and HUVEC were plated onto Matrigel, lumen structure of vascular network were formed after 6 hours of plating (Figure 2A). There were no statistical differences in the tube formation ability between hiPSC-1-EC, hiPSC-2-EC, hESC-EC and BM-EC (Figure 2A, all *P>0.05*).

Migration of EC through the endothelial lining is a key step in angiogenesis and vascular repair. Therefore, the migration ability of the hiPSC-1-EC, hiPSC-2-EC, hESC-EC and BM-EC was compared using the modified Boyden chamber assay. As compared with HUVEC and their corresponding controls with medium alone, hiPSC-1-EC, hiPSC-2-EC, hESC-EC and BM-EC showed comparable enhancement in migration ability with VEGF as the endothelial specific chemo-attractant (*P<0.05*). However, there were no significant differences in their migration ability among the five groups (Figure 2B, all *P<0.05*).

### Paracrine Secretion of Derived EC

**Figure 3:**
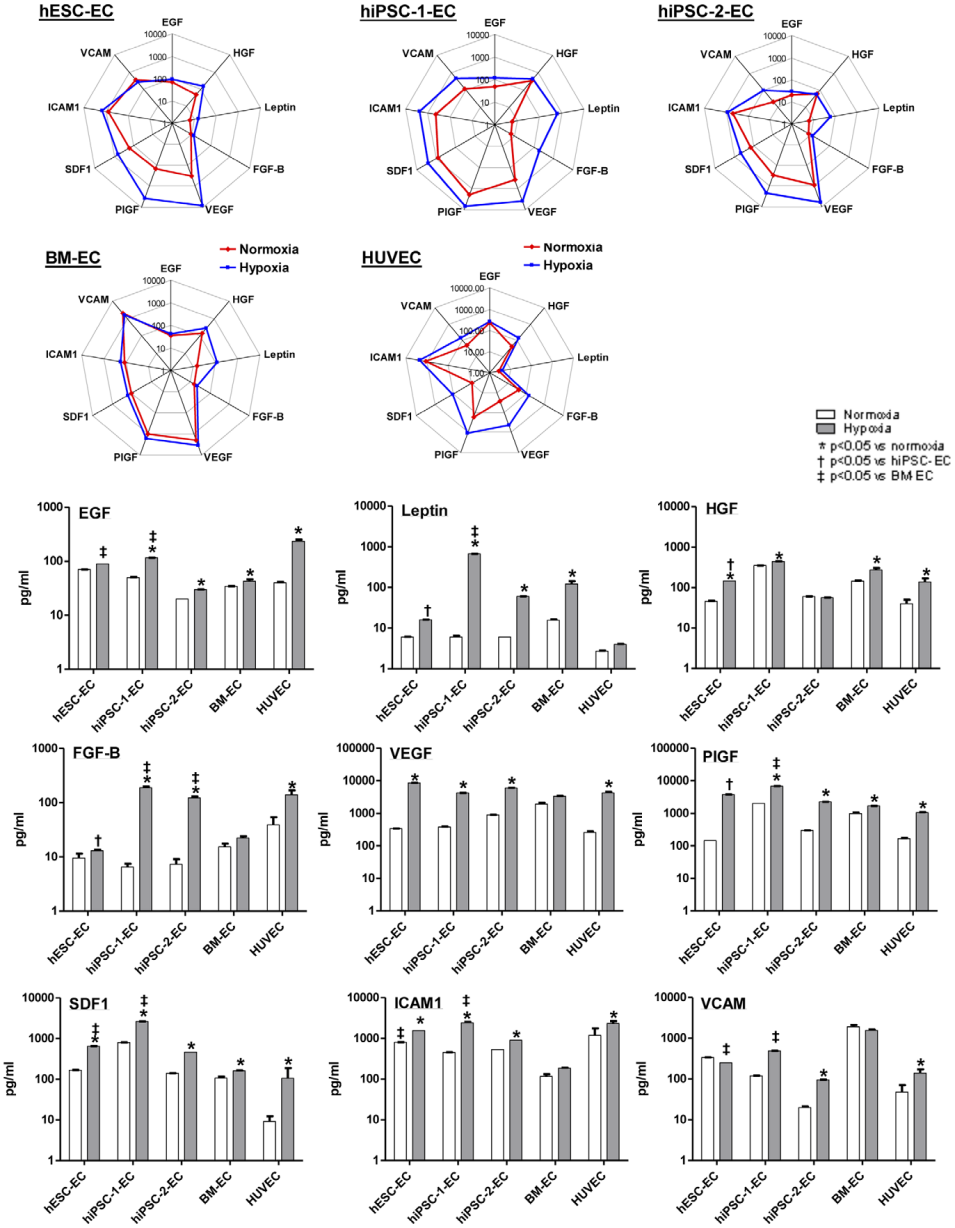
Angiogenic cytokine profiles of conditioned medium obtained from derived endothelial-like cells under normoxic and hypoxic condition. A total of 1×10^5^ cells were plated into 6-well gelatin coated plate for overnight incubation, then the cells were washed and cultivated in EBM2 basal medium without supplement for 5 days under normoxic (20% O_2_) or hypoxic (0.1% O_2_). Cytokine profiling of their conditioned mediums obtained were determined by Procarta multiplex bead-based immunoassay kit. (A) Bar charts and (B) multivariate radar chart showed the expression of epidermal growth factor (EGF), basic fibroblast growth factor (FGF-B), hepatocyte growth factor (HGF), leptin, vascular endothelial growth factor (VEGF), placental growth factor (PIGF) and stromal derived factor-1 (SDF-1); adhesion molecules including, ICAM1 and vascular cell adhesion molecule (VCAM) in the conditioned medium upon normoxic and hypoxic treatment.

The cytokine secretion profiles of EC derived from different sources were also investigated to provide insight into any potential difference in their paracrine effects after transplantation [17]. The cytokine profiles were quantified from the conditioned medium derived from hiPSC-1-EC, hiPSC-2-EC, hESC-EC, BM-EC and HUVEC using multiplex ELISA. During normoxic culture, similar pattern of cytokine expression (ICAM, VEGF, bFGF and leptin) were observed in the conditioned medium of hiPSC-1-EC, hiPSC-2-EC and hESC-EC, except that higher levels of HGF and PIGF were detected in hiPSC-1-EC and hiPSC-2-EC (Figure 3A). The conditioned medium of BM-EC had significantly higher levels of VEGF and VCAM and lower levels of ICAM1 than hESC-EC (all *P<0.05*, Figure 3A). Furthermore, the conditioned medium of BM-EC had significantly higher levels of VCAM and lower levels of SDF1 than hiPSC-1-EC and hiPSC-2-EC (all *P<0.05*, Figure 3A). The conditioned medium of the HUVEC had significantly lower levels of leptin and SDF-1 (Figure 3A).

To mimic the micro-environment in the ischemic tissue, hiPSC-1-EC and hiPSC-2-EC, hESC-EC, BM-EC and HUVEC were subjected to hypoxic stress. Multiplex ELISA revealed that the expression of major angiogenic factors including EGF, HGF, VEGF, PIGF and SDF-1 were significantly up-regulated in hiPSC-1-EC, hiPSC-2-EC, hESC-EC, BM-EC and HUVEC under hypoxia as compared with normoxia (all *P<0.05*, Figure 3A and B). Multivariate radar chart analysis showed that the magnitude of cytokine up-regulation upon hypoxic induction is more dramatic in hiPSC-1-EC and hiPSC-2-EC and hESC-EC compared with BM-EC (Figure 3B).

### Functional Improvement of Blood Flow in Ischemic Hind-limbs after Transplantation

**Figure 4:**
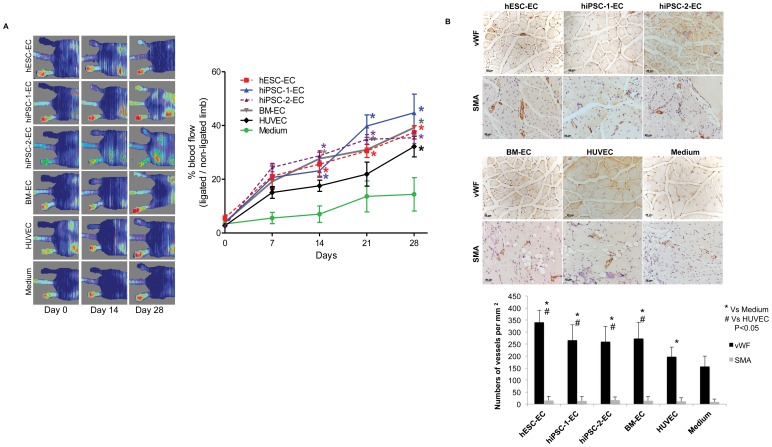
Improvement of blood perfusion in ischemic hind-limbs after transplantation of derived endothelial-like cells. (**A**) Laser Doppler scanning was preformed immediately after ligation of femoral artery to confirm the induction of hind-limb ischemia. Then repeated Doppler scanning was performed at Day 7, 14, 21 and 28 after transplantation of 3 million hESC-EC, two different hiPSC-ECs, BM-EC, HUVEC or medium alone into the ischemic muscle. Representative photos from the laser Doppler scanning at Day 0, Day 14 and Day 28 were shown. Blue color represents lower perfusion and red color represents higher perfusion (upper panel). In each animal, limb perfusion was quantified and the average of the perfusion ratio against the control limb was calculated in different time point (lower panel). As compared with medium injection, there was progressive and significant improvement of blood flow over the ischemic limbs after hESC-EC, hiPSC-ECs, BM-EC and HUVEC transplantation (n = 6 in each group, *p<0.05 vs. medium control at different time points). However, there were no significant differences in the blood flow over the ischemic limbs among those mice after hESC-EC, hiPSC-EC and BM-EC transplantation (p>0.05 at different time points). (**B**) Representative photos show the immunohistochemical staining for von Willebrand factor (vWF) and smooth muscle actin (SMA) of histological sections over the ischemic limbs at Day 28 after hESC-EC, hiPSC-EC, BM-EC or medium injection (upper panel). The number of vessels with positive staining (brown color) for vWF and SMA were determined under a light microscope with ×400 magnification with 20 random fields. As compared with medium injection, there were significant higher number of vessels with vWF positive but not SMA over the ischemic limbs after hESC-EC, hiPSC-ECs and BM-EC transplantation (*p vs. medium control and * vs HUVEC, p<0.05). However, there were no significant differences in the number of vessels with vWF and SMA positive over the ischemic limbs among those mice after hESC-EC, hiPSC-EC and BM-EC transplantation (p>0.05).

The therapeutics efficacy of hiPSC-1-EC, hiPSC-2-EC, hESC-EC, BM-EC and HUVEC for therapeutic angiogenesis was compared in a mouse model of hind-limb ischemia. After the femoral artery ligation, the blood flow of the ischemic limb decreased to only 3.7±0.4% of that the non-ischemic control limbs, confirming the successful induction of acute hind-limb ischemia. From Day 7 onward, there was significant and progressive improvement of ischemic limb blood flow in all groups of mice receiving EC transplantation as compared with medium group (Figure 4A, all *P<0.05*). At Day 28, mice treated with hiPSC-1-EC (44.8±6.9%), hiPSC-2-EC (38.2%±2.6%), hESC-EC (37.4±2.5%), BM-EC (39.2±5.8%) and HUVEC (29.6%±4.9%) transplantation had significantly higher hind-limb blood flow than the medium group (14.4±6.2%, all *P<0.05*)(Figure 4A). Nonetheless, there were no significant differences in the blood flow of ischemic limb between those mice treated with hiPSC-1-EC, hiPSC-2-EC, hESC-EC and BM-EC throughout the study period (all p<0.05), and their rate of blood flow improvement were superior to the HUVEC group from Day 0 to 14 (Figure 4A).

Immunohistological staining for vWF in the ischemic limbs at Day 28 demonstrated increased in capillary density after hiPSC-1-EC, hiPSC-2-EC, hESC-EC and BM-EC transplantation as compared with HUVEC group and control media injection (Figure 4B, all *P<0.05*). On the other hand, immunostaining for α-smooth muscle actin failed to reveal any difference in the numbers of arteries among different groups (Figure 4B, *P* *<0.05*), suggesting the development of angiogenesis rather than arteriogenesis after EC transplantation.

**Figure 5:**
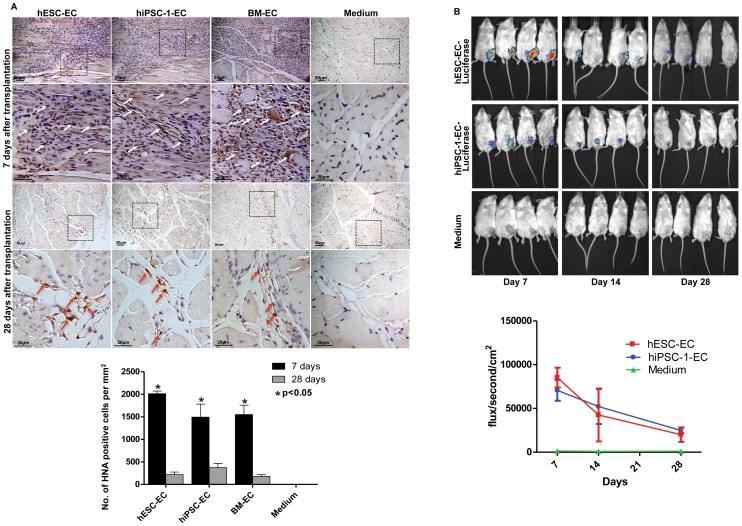
Retention of derived endothelial-like cells at Day 7, 14 and 28 after transplantation. (**A**) Representative photos show the immunohistochemical staining for human nuclear antigen (HNA) over the ischemic hind-limb at Day 7 and 28 after hESC-EC, hiPSC-1-EC, BM-EC or medium injection (upper panel). Red arrow indicated engrafted hESC-EC, hiPSC-EC, BM-EC in the ischemic limb with HNA positive (brown), indicating those cells were from human origin. At Day 7, clusters of HNA positive cells were observed at the injection sites after hESC-EC, hiPSC-1-EC, and BM-EC transplantation but not after medium injection. At Day 28, only a few HNA positive cells were found around the vasculature at the injection sites after hESC-EC, hiPSC-1-EC, and BM-EC transplantation. As compared with Day 28, the numbers of HNA positive cells detected at the injection sites were significantly higher at Day 7 after hESC-EC, hiPSC-1-EC, BM-EC transplantation (*p<0.05, lower panel). (**B**) Representative photos showing live cells tracking by quantifying luciferase activity using the Xenogen’s *in vivo* optical imaging systems at Day 7, 14 and 28 after hESC-EC, hiPSC-1-EC, or medium injection (upper panel). Luciferase activity was plot as total flux measurement in photons/second/cm^2^. Declined in intensity of luciferase activity over time after hESC-EC and hiPSC-1-EC transplantation was shown, suggesting progressive cell loss (n = 4 in each group, lower panel).

Histological examination did not reveal any teratoma formation at the transplanted sites in any of the mice after EC transplantation. Cell retention of transplanted EC was also examined at Day 7 and Day 28 in a subgroup of animals by immunohistological staining for HNA in the ischemic limbs. Comparing the number of HNA positive cells at Day 7 after transplantation, minimal transplanted cell retention was observed in all three groups of mice after EC transplantation as indicated by the markedly reduced number of HNA positive staining in ischemic limb harvested on Day 28 (Figure 5A).

To further evaluate the cell retention level and the proliferative ability of hiPSC-EC, *in- vivo* live cells imaging was employed to detect and quantify the emitted bioluminescence from luciferase-labeled hESC-EC and hiPSC-EC progeny. According to bioluminescence emitted from labeled progeny, hESC-EC and hiPSC-EC could be detected at Day 7 after transplantation and then started to decline. Nevertheless, transplanted hESC-EC and hiPSC-EC were still detected at Day 28 after transplantation with a gradual drop in signal intensity (Figure 5B).

## Discussion

Currently, BM-derived stem cells are the most common cell type investigated in clinical trials for treatment of cardiovascular diseases [3], [4], [18]. However, in order to overcome the scarcity and functional impairment of autologus BM stem cells, EC derived from pluripotent stem cells, such as hESC have been proposed as an alternative and unlimited source of cells for therapy. The latest advances in the generation of hiPSC [10] has shed light into the field of cell-based therapy by providing an alternative unlimited source for autologous transplantation which can potentially solve the problem of availability [19]. In the current study, we sought to evaluate the therapeutic efficacy of EC derived from different cell sources in treatment of ischemic cardiovascular diseases.

Here, we confirmed that one hESC line and two hiPSC lines could be differentiated into functional EC which express a comparable level of endothelial markers as BM-EC and HUVEC. *In-vitro* tube formation and cellular migration assays revealed that hESC-EC and hiPSC-EC have similar angiogenic function as BM-EC and HUVEC. Although there was some discrepancy in the angiogenic cytokine profile in the conditional medium between hiPSC-EC, hESC-EC, BM-EC and HUVEC at baseline, significant increased in the level of multiple major angiogenic cytokines, including EGF, HGF, VEGF, PIGF and SDF-1 were observed in the conditioned medium derived from all type of EC after hypoxia. Moreover, hiPSC-EC and hESC-EC had even greater response to hypoxic induction in their expression of angiogenic cytokines than the BM-EC and HUVEC. These angiogenic cytokines have been shown to enhance neovascularization in the ischemic sites [20].

Therefore, the increased expression of these angiogenic factors from the transplanted EC upon hypoxia in the ischemic condition of the hind-limb could provide paracrine effect to enhance neovascularization.

In concordance with these observations, our results also demonstrated a similar overall therapeutic efficacy of EC derived from hiPSC, hESC and BM-MNC as determined by laser Doppler blood flow up to Day 28 in the mouse model of hind-limb ischemia. Despite progressive improvement of blood flow over the ischemic limbs was observed in serial laser Doppler blood flow measurement, immunohistochemical staining and luciferase assay revealed only very small number of the EC remained and engrafted into the vasculature at the transplanted ischemic limbs. Therefore, majority of the beneficial effects exert by the transplanted EC is likely to be mediated via other mechanisms, such as paracrine secretion rather than *de-novo* formation of vasculature by the transplanted EC [1], [21], [22]. Furthermore, we only observed increased angiogenesis rather than arteriogenesis after EC transplantation.

Our results also confirmed previous findings [23] and showed that HUVEC cells have a lower capacity in improving blood flow and neovascularization in the hind-limb ischemic mouse model than those EC derived from BM, hESC or hiPSC. This finding suggests that the paracrine function of HUVEC is less effective than EC derived from other cell sources for enhancing neovascularization.

Since the initial description of successful generation of hiPSC from adult fibroblast, several clinical compliance protocols had been developed, such as the use of xeno-free and feeder-free system [14] and the generation of hiPSC from donor cells that does not require an invasive procedure, e.g. urine cells [24] and hair follicles [25]. Moreover, recent breakthrough of generating hiPSC using mRNA transfer [26] without the need of viral mediated transfer has brought the hiPSC technology closer to the clinic.

To our knowledge, this study is the first to compare the *in vitro* and *in vivo* therapeutic efficacy of EC derived from hiPSC, hESC, BM and normal human endothelial cells. Although hiPSC-EC only provide a similar therapeutic efficacy as BM-EC and hESC-EC, the use of patients-specific hiPSC-EC can circumvent issues related to the clinical use of hESCs, as well as the need for an invasive procedure for harvesting an adequate amount of BM cells for transplantation. In this study, ECs with similar functional characteristics and therapeutic potential could be derived from two different hiPSC lines. Importantly, we did not observe any teratoma formation in mice after transplantation of both hESC-EC or hiPSC-EC, suggesting that the risk of uncontrolled proliferation or growth from those EC derived from pluripotent stem cells is low. Nevertheless, this finding needs to be further confirmed by future studies.

In this study, only 1 out of 6 specimens of BM mononuclear cells from patients with severe coronary artery disease could be able to differentiate into functional EC for transplantation due to their poor survival and proliferation capability as shown in previous studies [8]. Furthermore, the underlying concomitant conditions of these patients, such as heart failure, hypertension and diabetes [5]–[7] may also offset the function of their BM stem cells. The observation also highlight that autologous BM-EC transplantation might be feasible and effective in a small number of patients with cardiovascular diseases. The low successful rates to obtain functional EC from BM cells as well as their larger variability in characteristics are major limitations for their clinical use. Our results from EC derived from two hiPSCs and one hESC consistently demonstrated that they are not inferior to BM-EC for both *in-vivo* and *in-vitro* neovascularization. Importantly, functional EC could be derived from existing hESC and hiPSC lines despite multiple passages. Thus, these cell types are more stable sources of functional EC for potential therapeutic applications.

Taken together, our results showed that functional EC could be generated from hESC and iPSC with similar therapeutic efficacy for attenuation of severe hind-limb ischemia. Differentiation of functional BM-EC was more difficult to achieve in patients with cardiovascular diseases, and hESC-EC or iPSC-EC are readily available as “off-the-shelf” format for treatment of tissue ischemia.

This study has limitations. First, the precise mechanism of therapeutic benefit for each of the cell types remains unknown. In this study, we showed that only very small numbers of transplanted cells were detected at Day 28. This finding suggests that paracrine actions of the transplanted ECs are the likely mechanisms that contribute to increased angiogenesis. On the other hand, whether those transplanted ECs directly contribute to the paracrine effects is unknown, as other mechanisms such as recruitment of endogenous local or circulating progenitor cells into the ischemic tissues are not investigated in this study. Second, the precise role of different cytokines secreted by the transplanted cells remains unclear and only limited panel of cytokines were tested in this study.
